# Squamous Cell Carcinoma of the Nail Unit: A Comprehensive Review of Clinical Features, Diagnostic Workflow, Management Strategies and Therapeutic Options

**DOI:** 10.3390/diagnostics15182378

**Published:** 2025-09-18

**Authors:** Federico Venturi, Elisabetta Magnaterra, Biagio Scotti, Aurora Alessandrini, Leonardo Veneziano, Sabina Vaccari, Carlotta Baraldi, Emi Dika

**Affiliations:** 1 Department of Medical and Surgical Sciences (DIMEC), Alma Mater Studiorum, University of Bologna, 40126 Bologna, Italy; elisabetta.magnaterra@unibo.it (E.M.); biagio.scotti2@unibo.it (B.S.); emi.dika3@unibo.it (E.D.); 2 Oncologic Dermatology Unit, IRCCS Azienda Ospedaliero-Universitaria di Bologna, 40138 Bologna, Italy; aurora.alessandrini@aosp.bo.it (A.A.); leonardo.veneziano@aosp.bo.it (L.V.); sabina.vaccari@aosp.bo.it (S.V.); carlotta.baraldi@aosp.bo.it (C.B.)

**Keywords:** squamous cell carcinoma, nail cancer, dermoscopy, onychoscopy, confocal microscopy, molecular pathology, HPV-associated carcinoma, nail surgery, Mohs micrographic surgery

## Abstract

**Background/Objectives:** Squamous cell carcinoma of the nail unit (SCCNU) is a rare yet often underrecognized malignancy that can lead to delayed diagnosis and significant functional morbidity. This review aims to comprehensively summarize the current understanding of SCCNU, focusing on its clinical, dermoscopic, and molecular features, diagnostic approaches, and evolving management strategies, including the role of emerging technologies and immunotherapy. **Methods:** A detailed literature review was conducted using peer-reviewed publications, case series, and institutional guidelines related to SCCNU. Emphasis was placed on studies addressing clinical presentation, dermoscopic patterns, molecular pathology, histologic subtypes, imaging, biopsy techniques, staging systems, and both conventional and novel therapeutic approaches. Comparative analyses of histopathological variants and diagnostic algorithms were included. **Results:** SCCNU presents in patients with diverse clinical manifestations, often mimicking benign nail disorders, leading to diagnostic delays. Dermoscopy improves lesion visualization, revealing features such as vascular changes and onycholysis. Histologically, SCCNU exhibits two main subtypes: basaloid (HPV-related) and keratinizing (HPV-negative) types. Molecular analyses have identified TP53 as the most frequently mutated gene, with additional alterations in HRAS, BRAF, and TERT. Imaging modalities such as MRI and LC-OCT aid in staging and surgical planning. Management is centered on complete excision—often via Mohs micrographic surgery—while topical, intralesional, and HPV-directed therapies are under investigation. Immunohistochemical markers (p16, Ki-67, AE1/AE3) and neoadjuvant immunotherapy represent promising adjuncts. **Conclusions:** Early diagnosis through non-invasive imaging, improved molecular characterization, and personalized treatment strategies are essential to advancing care in SCCNU. Future directions include clinical trials evaluating immunotherapy, vaccine strategies, and precision-guided surgical approaches to preserve function and minimize recurrence.

## 1. Introduction

Squamous cell carcinoma (SCC) is the second most common form of skin cancer, arising from the malignant proliferation of keratinocytes [1]. While SCC typically affects sun-exposed areas such as the head and neck or the extremities, it can also develop in specialized anatomical sites like the nail unit. Squamous cell carcinoma of the nail unit (SCCNU) is a rare but clinically significant subtype of cutaneous SCC, constituting a diagnostic and therapeutic challenge due to its subtle presentation, potential for misdiagnosis, and risk of invasive growth [2].

SCCNU often originates from the nail bed or matrix and typically presents as a slowly growing, locally aggressive tumor [2]. Because of the close anatomical relationship between the nail unit and the underlying phalanx, even small tumors may cause significant functional impairment or bone invasion if not recognized early. Despite its rarity, SCC is the most common primary malignancy of the nail apparatus, far outnumbering other conditions like melanoma, basal cell carcinoma, and adnexal tumors in this location [3].

Early lesions often resemble benign nail disorders, such as chronic paronychia, warts, onychomycosis, traumatic dystrophy, or pyogenic granulomas. As a result, diagnosis is commonly delayed by several months or even years, with studies reporting average diagnostic delays ranging from 12 to 36 months [4]. This delay is particularly concerning as it increases the risk of local invasion, destruction of the distal phalanx, and, in rare cases, regional or distant metastasis [2]. Hence, early recognition, clinical vigilance, and timely biopsy are critical to improving patient outcomes.

In recent years, dermoscopy and onychoscopy have emerged as valuable non-invasive tools, enabling clinicians to identify suspicious vascular and keratinous patterns not visible to the naked eye [5,6,7,8,9]. Newer imaging technologies, such as reflectance confocal microscopy (RCM) and line-field confocal optical coherence tomography (LC-OCT), are under investigation for their ability to guide biopsy and surgical planning [10,11]. Histopathology remains the gold standard, but immunohistochemistry and molecular analyses now provide additional insights into tumor biology [2]. Notably, two distinct histological and molecular subtypes have been described: the HPV-related basaloid variant and the keratinizing HPV-negative form. Mutations in genes such as TP53, HRAS, and BRAF have further enriched our understanding of pathogenesis [12,13].

In parallel, the therapeutic landscape of SCCNU is evolving. Mohs micrographic surgery (MMS) is now regarded as the gold standard for localized disease, providing superior margin control and functional preservation [14]. For more advanced cases, wide local excision or, when bone is involved, partial amputation may be necessary. Adjunctive treatments, including radiotherapy, topical or intralesional therapies, and systemic immunotherapies, are increasingly being explored in selected patients. Furthermore, the role of HPV vaccination and targeted therapies is an area of active research, raising the possibility of personalized treatment approaches in the near future [15].

Given these developments, a comprehensive synthesis of clinical features, diagnostic strategies, molecular insights, and therapeutic options for SCCNU is warranted. The aim of this review is therefore to summarize current knowledge, highlight practical diagnostic and management approaches for clinicians, and discuss emerging technologies and future directions in the care of patients with this rare but impactful malignancy.

## 2. Materials and Methods

**Figure 1:**
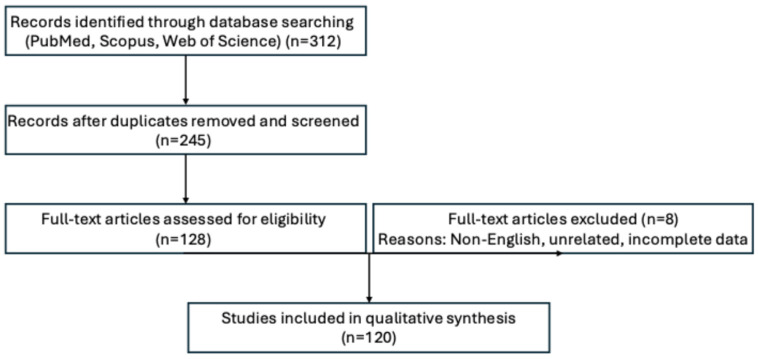
Flow diagram illustrating the study selection process for this review on squamous cell carcinoma of the nail unit (SCCNU). A total of 312 records were identified through electronic databases (PubMed, Scopus, Web of Science). After removal of duplicates, 245 articles were screened by title and abstract. Of these, 128 full-text articles were assessed for eligibility, with 8 excluded due to irrelevance, incomplete data, or non-English language. In total, 120 studies were ultimately included in the qualitative synthesis.

A comprehensive literature search was performed using databases including PubMed, Scopus, and Web of Science up to July 2025. Keywords used included “nail unit squamous cell carcinoma”, “SCC nail”, “dermoscopy”, “molecular features”, “HPV and nail cancer”, “immunohistochemistry”, and “management of SCCNU”. Articles selected included original research, systematic reviews, clinical guidelines, case reports, and expert opinions relevant to diagnosis, staging, histological variants, molecular pathology, imaging, and both surgical and non-surgical management. Priority was given to peer-reviewed studies published in English. Reference lists of included articles were also reviewed to identify additional pertinent sources (Figure 1). This review aimed to integrate recent findings with current clinical practice to inform early diagnosis and personalized management approaches for SCCNU.

## 3. Anatomy & Epidemiology

The nail unit is a specialized skin appendage composed of several interrelated structures that work together to produce and support the nail plate [16,17,18]. These include the following:

- Nail Matrix: Located beneath the proximal nail fold, the matrix is responsible for producing the bulk of the nail plate. Tumors arising in this area can significantly disrupt nail growth and morphology.
- Nail Bed: A thin epithelial layer lying beneath the nail plate that continues to contribute to nail formation and maintains nail adherence. The nail bed is a common origin site for subungual squamous cell carcinoma.
- Nail Plate: The hard, keratinized structure composed primarily of compacted keratinocytes. It serves as a protective covering but also conceals early subungual tumors, contributing to diagnostic delay.
- Hyponychium: The epithelium under the distal nail plate, located just beyond the nail bed, which acts as a barrier to pathogens.
- Proximal and Lateral Nail Folds: These surround and support the nail plate, providing a reservoir for inflammatory and neoplastic processes.

The intimate anatomical relationship between these structures and the underlying bone (distal phalanx) means that any malignant process can quickly involve osseous tissue if not diagnosed and managed promptly. SCCNU typically arises from the nail bed or lateral nail fold, though it may occasionally originate in the nail matrix or hyponychium [2]. Because of the confined anatomical space and delayed presentation, even small tumors can exert significant destructive potential, invading adjacent structures or underlying bone with relative ease.

### 3.1. Epidemiology

Although squamous cell carcinoma is one of the most common forms of cutaneous malignancy, SCCNU is considered rare, accounting for less than 2% of all hand malignancies and a similarly small percentage of cutaneous SCCs overall [1,19]. However, it is the most frequently diagnosed malignant tumor of the nail apparatus, surpassing other entities such as melanoma, basal cell carcinoma, and adnexal carcinomas in this specific location.

The true incidence of SCCNU is not well established due to its rarity and frequent underdiagnosis. However, the condition predominantly affects middle-aged and elderly individuals, with the peak incidence reported between the ages of 50 and 70 years. Cases have been documented in both younger and older patients, especially in the context of risk factors such as HPV infection or immunosuppression [20].

- Sex distribution: Historically, SCCNU has been reported more commonly in males, particularly when affecting the fingernails. This has been attributed to greater exposure to occupational trauma, ultraviolet radiation, and chemical carcinogens [21]. However, some recent studies suggest a narrowing gender gap, possibly due to increased awareness and broader occupational exposure among females.
- Site predilection: The thumb and index finger are the most frequently involved digits, likely due to their greater exposure to trauma and environmental factors [22]. SCC of the toenails is rarer but not unheard of, and it often goes unrecognized longer due to lower patient concern or visibility.

### 3.2. Risk Factors

Several etiological and environmental factors have been associated with an increased risk of SCCNU:

- Chronic trauma or irritation: Repeated mechanical trauma, especially in manual laborers, can lead to persistent inflammation and epithelial damage, fostering carcinogenesis [23,24,25]. Moreover, epithelial trauma can expose basal keratinocytes to HPV virions.
- HPV: High-risk HPV types, particularly HPV-16, have been implicated in the pathogenesis of SCCNU. Studies have detected HPV DNA in approximately 30–60% of SCCNU specimens [20,26,27]. The virus is believed to promote carcinogenesis via E6 and E7 oncoproteins that inhibit p53 and retinoblastoma protein (Rb), respectively [28]. The association between high-risk HPV infection and SCCNU has raised interest in potential public health interventions, including HPV vaccination. While current vaccines target HPV types most relevant to cervical and anogenital cancers, they may also offer indirect protection against HPV-related skin cancers, especially in immunosuppressed individuals or those with occupational exposure [29,30]. Further epidemiological studies are needed to assess the vaccine’s role in reducing SCCNU incidence.
- Immunosuppression: Organ transplant recipients, HIV-positive individuals, and patients on chronic immunosuppressive therapy exhibit a higher risk of developing SCC, including in the nail unit [31,32]. Immunosuppressed patients present with SCCNU at an earlier age and with a higher frequency of polydactylous disease [3]. Additionally, the nail matrix is an immune-privileged site, with reduced expression of HLA class I molecules (HLA-A, -B, -C), along with a relative paucity of CD4+ and CD8+ T lymphocytes. Furthermore, the antigen-presenting functions of Langerhans cells and macrophages may be diminished in this region, potentially impairing local immune surveillance. However, because most SCCNU arise from the nail bed or periungual folds, the precise contribution of the matrix’s immune-privileged status to HPV-related carcinogenesis remains unclear [3].
- Ionizing radiation and chemical carcinogens: Historical exposure to X-rays, arsenic compounds, and industrial solvents has been linked to increased risk [33,34,35].
- Smoking and UV exposure: Though less directly implicated in nail carcinogenesis than in other forms of SCC, these factors may still contribute, particularly in patients with field cancerization of surrounding skin [36,37].
- Pre-existing conditions: Long-standing chronic infections (e.g., verruca vulgaris and paronychia), actinic keratoses, or genetic conditions such as xeroderma pigmentosum may predispose to SCCNU [2].

One of the key epidemiological challenges in SCCNU is the delay in diagnosis. Studies report a mean diagnostic delay of 12–36 months, often because the lesion is mistaken for a benign condition such as a fungal infection, chronic dermatitis, or wart. Misdiagnosis is particularly common in cases with minimal visible nail plate changes or when the lesion is obscured by the nail plate itself. As a result, by the time of diagnosis, many cases have already progressed to involve the bone or soft tissue, necessitating more aggressive surgical intervention, including partial or complete digit amputation [38,39].

## 4. Clinical Presentation

SCCNU presents a wide spectrum of signs and symptoms, often overlapping with benign nail disorders [40,41]. The variable clinical morphology and rarity of the disease contribute to diagnostic delays, with many patients undergoing ineffective treatments for presumed infections or inflammatory conditions before a biopsy is performed (Table 1). SCCNU typically presents in one of the following forms [2]:

- Subungual hyperkeratosis: Accumulation of keratin debris beneath the nail plate, often associated with onycholysis (separation of the nail plate from the bed).
- Nail dystrophy: Irregular growth, longitudinal ridging, or splitting of the nail.
- Warty or verrucous lesion: Particularly when the tumor extends beyond the nail bed or into the lateral fold.
- Ulceration or erosion: Advanced lesions may show destruction of overlying tissues and nail plates.
- Persistent pain or bleeding: Especially when the tumor invades deeper structures or erodes vessels.
- Mass formation: A firm, nodular lesion under or around the nail is sometimes visible only after nail removal.

These features are generally unilateral and confined to a single digit, with the thumb and index finger being most affected, likely due to increased exposure to trauma or environmental insults. Some cases may present as pigmented lesions, mimicking subungual melanoma [8,9]. Others can be amelanotic, presenting only as a pink or reddish nodule under the nail plate. Rarely, paronychia-like presentations with swelling and erythema of the proximal nail fold may lead to misdiagnosis, such as chronic bacterial or fungal infections.

### 4.1. Red Flags Suggesting SCCNU

Certain clinical features should raise suspicion of SCCNU, particularly when they persist despite appropriate therapy [42,43]:

- Chronic, non-healing nail lesions are unresponsive to antifungals or antibiotics.
- A solitary, progressive subungual mass or growth.
- Intermittent or spontaneous bleeding from the nail or surrounding tissue.
- Nail plate destruction without a history of significant trauma.
- Sudden onset of nail dystrophy in a middle-aged or elderly patient.
- Associated pain or discomfort is not typical for fungal infections or benign growth.

In any of these scenarios, a biopsy is essential. Delay in performing a biopsy can lead to progression of disease, bony invasion, and more aggressive surgical management.

### 4.2. Dermoscopic Features

Dermoscopy (or onychoscopy, when applied to nails) has become an indispensable tool in the early detection and evaluation of nail unit tumors [44]. For SCCNU, dermoscopy bridges the gap between clinical uncertainty and the decision to biopsy, especially in ambiguous or chronic presentations. Its utility lies in identifying vascular, keratinous, and pigment-related patterns that are not visible to the naked eye. Given the potential of SCCNU to mimic benign conditions, dermoscopy enhances diagnostic accuracy by revealing morphologic signs of malignancy (Table 2 and Table 3) [2,7,45]. Nevertheless, while dermoscopy provides vital diagnostic clues, it does not replace histopathological confirmation.

While dermoscopy improves diagnostic confidence, its limitations include the following: overlap with inflammatory and infectious nail disorders, operator dependence, variability in interpretation, and difficulty in visualizing subungual lesions beneath an opaque nail plate.

### 4.3. Reflectance Confocal Microscopy and Line-Field Confocal Optical Coherence Tomography

Reflectance Confocal Microscopy (RCM and line-field confocal optical coherence tomography (LC-OCT) are non-invasive imaging technologies that have emerged as promising tools in the evaluation of nail unit lesions, including SCCNU. RCM allows for real-time, cellular-level imaging of superficial skin layers and can visualize architectural disarray, keratinocyte atypia, and abnormal vascular patterns without the need for biopsy [46]. Although limited by the nail plate’s opacity, RCM is useful in periungual and lateral fold lesions or after partial nail avulsion [11,47,48,49]. LC-OCT, a newer hybrid technology, combines the resolution of confocal microscopy with the depth penetration of OCT (~500 μm), enabling detailed cross-sectional and en-face views of the epidermis and superficial dermis. In SCCNU, LC-OCT can detect features such as hyperkeratosis, irregular epithelial projections, and keratin-filled invaginations consistent with squamous proliferation. These modalities can improve pre-biopsy triage, guide targeted sampling, and potentially monitor response to non-surgical therapies [10]. However, their role in routine practice remains investigational and dependent on further validation.

Therefore, in any case with suspicious findings, a biopsy of the nail bed or matrix is warranted. Dermoscopy also helps define the most representative biopsy site, particularly in diffuse lesions.

## 5. Histopathology and Immunohistochemistry

Accurate diagnosis of SCCNU requires histopathological confirmation, often supplemented by immunohistochemical (IHC) and molecular analyses. These approaches not only confirm the malignant nature of the lesion but also offer insight into its biological behavior, potential aggressiveness, and viral associations such as HPV. While the overall molecular landscape of SCCNU is still emerging, recent advances in sequencing and biomarker studies have shed light on its heterogeneity and pathogenesis.

### 5.1. Histopathology

Histologically, SCCNU demonstrates features typical of SCC arising in other skin sites, but with variations based on anatomical constraints and chronic inflammation associated with the nail apparatus. Typical histopathological features include acanthosis, keratinocyte atypia, dyskeratosis, keratin pearls, infiltrative nests, perineural invasion (PNI), and bone invasion. Specifically, in situ SCC is characterized by full-thickness atypia of the epithelium without dermal invasion, while invasive SCC shows penetration of the basement membrane and may involve soft tissue or bone. The final histopathological report should include histological risk factors that are relevant for the staging and prognosis, including the thickness, depth of invasion, the grade of differentiation, and margins status and desmoplastic type [50]. Nail matrix-origin tumors may be deceptively bland in early stages, emphasizing the importance of deep and well-oriented biopsy specimens [51].

### 5.2. Immunohistochemistry

Immunohistochemistry (IHC) is useful in confirming squamous differentiation, evaluating proliferation, and identifying specific markers associated with tumor aggression and potential for metastasis [52]. Its role is crucial in the diagnostic work-up, particularly in histologically ambiguous or poorly differentiated tumors. It assists in confirming squamous epithelial lineage, assessing proliferative behavior, evaluating viral oncogenesis, and ruling out histologic mimics such as melanoma or adnexal tumors [53]. Pan-cytokeratin markers AE1/AE3 are foundational in epithelial tumor diagnosis. These broad-spectrum antibodies detect a wide range of keratin subtypes expressed in simple and stratified epithelium [54]. In SCCNU, they show strong cytoplasmic positivity, confirming epithelial derivation and helping exclude mesenchymal or melanocytic tumors. Moreover, diffuse p63 positivity is particularly helpful in distinguishing SCC from basal cell carcinoma or adnexal neoplasms [55]. Epithelial membrane antigen (EMA) is another important epithelial marker expressed on the apical surface of many glandular and squamous epithelia. In SCCNU, EMA demonstrates granular cytoplasmic or membranous positivity, supporting epithelial origin and helping distinguish SCC from melanoma or poorly differentiated sarcomas. Though less specific than p63, EMA complements broader panels, especially in high-grade or ambiguous tumors [56]. Ki-67, a nuclear marker of cellular proliferation, is frequently used to evaluate tumor growth kinetics. In SCCNU, an elevated Ki-67 index (>20–30%) indicates high mitotic activity and correlates with poor differentiation or deeper invasion, assisting in prognostication and grading [53]. In tumors suspected to be HPV-related, p16 IHC is employed as a surrogate marker for high-risk HPV integration, particularly HPV-16. Diffuse, strong nuclear and cytoplasmic p16 expression is suggestive of viral oncogenesis, often seen in younger patients or verrucous variants, and may guide therapeutic decision-making or indicate eligibility for HPV-specific interventions [57]. Lastly, podoplanin (PDPN, D2-40) is frequently overexpressed in invasive SCC, including SCCNU, and highlights tumor-stroma interfaces and lymphatic channels. PDPN expression is associated with increased invasiveness, lymphatic dissemination, and potentially worse prognosis, making it useful in assessing aggressive or deeply infiltrative lesions [58]. Recent studies have described two primary histopathological subtypes of SCCNU, distinguished by their epithelial appearance under low-power microscopy and correlated with distinct clinical features [12,13]:

- The basaloid (blue) pattern: This subtype is composed predominantly of basaloid keratinocytes and exhibits two histologic variants. The first features a flat epithelium with keratinocytes showing scant cytoplasm, hyperchromatic basophilic nuclei, and frequent full-thickness mitoses. The second, a verrucous variant, contains keratinocytes with enlarged eosinophilic cytoplasm, prominent nuclear atypia, atypical hyperparakeratosis, and koilocytosis. This basaloid pattern has been strongly associated with abnormal p53 expression and the presence of high-risk HPV infection. Clinically, it is more often observed in SCC in situ, tends to occur in the periungual area, and is more common in younger patients.
- The keratinizing (pink) pattern: Characterized by a flat or ulcerated epithelium composed of keratinocytes with vesicular nuclei, eosinophilic cytoplasm, and marked dyskeratosis, this pattern gives the tissue a hypereosinophilic “pink” appearance under low magnification. Histologic features include elongation of rete ridges, loss of cellular cohesion, and the presence of cytoid bodies, but notably, koilocytosis is absent. The keratinizing pattern shows a significant association with elevated Ki-67 expression, reflecting high proliferative activity, and lacks evidence of HPV infection. It is typically seen in invasive SCC, usually located in the subungual region, and occurs predominantly in elderly individuals.

## 6. Molecular Features

Recent molecular profiling of SCCNU has begun to reveal a diverse and complex mutational landscape, though much work remains to be done. Data from next-generation sequencing (NGS) studies and HPV integration analyses suggest a multi-factorial origin [20]. In a genomic analysis of SCCNU, TP53 emerged as the most frequently mutated gene, present in approximately 30% of tumors, followed by less common mutations in HRAS, BRAF, TERT, and GNA11, each identified in at least two cases [20]. Notably, no clear correlation was observed between HPV status and specific mutational profiles. Compared to cutaneous SCC, nail unit SCCs exhibit a lower overall mutational burden. While cutaneous SCCs frequently display ultraviolet (UV)-induced mutations, particularly in genes like TP53, NOTCH1, and NOTCH2, such UV mutation signatures were detected in only 20% of SCCNU, all of which originated from the nail fold, a partially UV-exposed site [20,59,60]. The difference is attributed to the UV-shielding properties of the nail plate, which blocks nearly all UV-B radiation and allows minimal UV-A transmission [61]. These findings highlight a key molecular distinction between SCCNU and cutaneous SCCs [62]: the absence of UV-driven mutagenesis in most SCCNU supports the theory of alternative, non-UV carcinogenic pathways. Other recurrent, though less frequent, mutations include EGFR, CTNNB1 (β-catenin), c-KIT, DICER1, and GNAS, suggesting possible involvement of tyrosine kinase signaling, Wnt/β-catenin pathway dysregulation, and epigenetic modulation via microRNA processing [20]. Emerging research indicates that immune evasion mechanisms may also play a role in SCCNU progression. Tumor microenvironment profiling has identified increased expression of PD-L1 and downregulation of MHC class I molecules, both of which may enable immune escape, though these features have not yet been extensively studied in the nail unit specifically [63]. As such, molecular profiling may eventually guide targeted therapies—such as EGFR inhibitors in EGFR-mutated tumors or immunotherapy in HPV-driven or PD-L1–positive SCCNU—though current evidence remains preliminary [64].

## 7. Diagnostic Workflow

**Figure 2:**
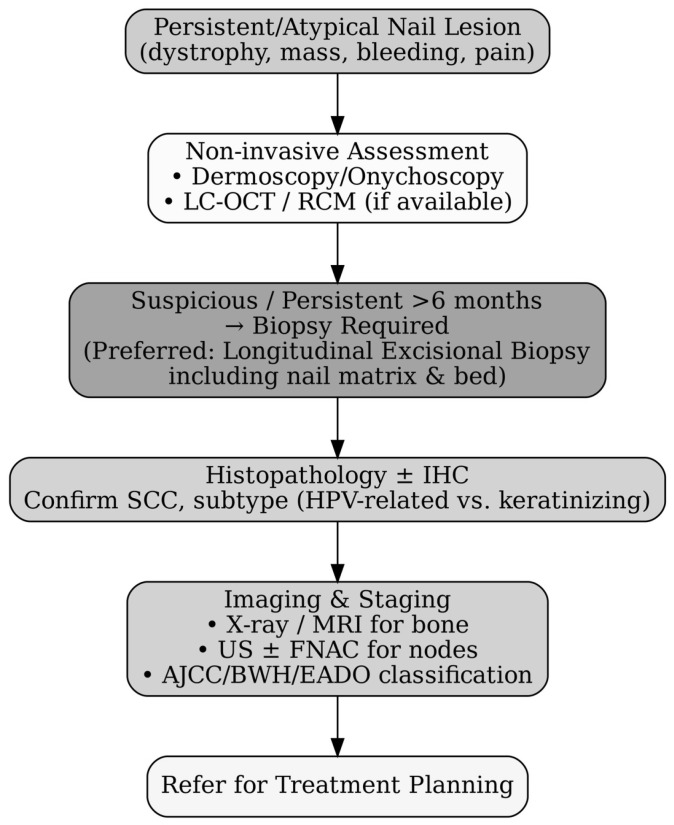
Diagnostic workflow for squamous cell carcinoma of the nail unit (SCCNU). Algorithm illustrating the recommended diagnostic pathway from initial clinical suspicion to treatment referral. Persistent or atypical nail lesions should first undergo non-invasive evaluation with dermoscopy/onychoscopy or advanced imaging where available (RCM/LC-OCT). If findings remain suspicious or lesions persist beyond six months, biopsy is mandatory. A longitudinal excisional biopsy that includes both the nail matrix and bed is preferred for optimal histological assessment. Histopathology ± immunohistochemistry confirms diagnosis and subtype, followed by appropriate imaging and staging to guide management.

Timely diagnosis of SCCNU is critical to prevent delayed treatment and disease progression. Given its rarity and clinical mimicry of benign nail disorders, SCCNU often remains undetected until advanced stages. A systematic and stepwise diagnostic approach is therefore essential to distinguish it from inflammatory, infectious, and other neoplastic nail conditions (Figure 2).

### 7.1. Imaging

Imaging assessment plays a critical role in the diagnostic evaluation, staging, and surgical planning of SCCNU, particularly when there is suspicion of deep tissue invasion, periosteal involvement, or recurrence. Due to the close anatomical relationship between the nail unit and the distal phalanx, early bone involvement may be clinically occult, making imaging essential for accurate assessment. Plain radiography (X-ray) is typically the first-line modality used to evaluate bony structures [65]. It can reveal periosteal reactions, cortical erosion, or osteolytic lesions, which are suggestive of advanced tumor invasion. However, its sensitivity is limited in detecting early marrow infiltration or soft tissue changes [66]. For superior soft tissue contrast and detailed visualization, magnetic resonance imaging (MRI) is the gold standard [67]. MRI is particularly valuable for delineating tumor boundaries, assessing nail matrix, bed, and fold involvement, and identifying bone marrow infiltration or perineural spread [68,69]. T1-weighted images can highlight bone involvement, while T2-weighted and STIR sequences are sensitive for detecting soft tissue edema, inflammatory changes, and tumor extent. In cases of ambiguous or recurrent disease, contrast-enhanced MRI can help differentiate tumor from post-surgical or inflammatory changes [13]. Ultrasound, particularly high-frequency Doppler ultrasound, is useful for assessing regional lymph nodes, providing information on nodal size, shape, cortical thickness, and vascularity [70,71,72,73]. Suspicious nodes may warrant fine-needle aspiration cytology (FNAC) under ultrasound guidance [74,75]. Computed tomography (CT) can be used to evaluate bony destruction with high spatial resolution and may assist in surgical planning, especially in advanced cases requiring amputation. CT is also helpful when MRI is contraindicated [76,77]. For systemic staging in high-risk or advanced SCCNU (e.g., with perineural invasion or recurrent disease), positron emission tomography–computed tomography (PET-CT) or whole-body CT may be considered to evaluate for regional nodal or distant metastases, although this is uncommon [78,79]. Additionally, emerging modalities such as line-field confocal optical coherence tomography (LC-OCT) and reflectance confocal microscopy (RCM) are being explored for superficial tumor margin assessment and may aid in pre-biopsy planning, although they remain investigational in nail unit tumors [80].

### 7.2. Biopsy Techniques

Biopsy is mandatory for diagnosis, especially in lesions persisting >6 months or showing atypical features. Several techniques are used depending on lesion location and extent [81,82]. Longitudinal excisional biopsy is generally considered the gold standard for nail unit tumors, as it allows comprehensive sampling from the nail matrix to the hyponychium, ensuring inclusion of potential tumor margins. This technique is especially useful when the lesion is diffuse or involves multiple components of the nail unit. In focal or well-demarcated lesions, a punch biopsy (typically 3–5 mm) may be appropriate, particularly when targeting a periungual nodule or keratotic plaque. However, caution is advised as superficial punch biopsies may miss deeper invasion, leading to false negatives or undergrading of the tumor. For superficial or in situ disease, a deep shave biopsy may suffice, though this technique may not reliably assess depth or margin status. When subungual involvement is suspected, partial or complete nail plate avulsion is often necessary to expose the underlying bed or matrix, facilitating accurate tissue sampling. Incisional biopsies are suitable for larger lesions where complete removal is not initially feasible; however, they must be deep enough to include dermis to evaluate for invasion. Proper orientation and tissue handling are essential to preserve architecture and guide surgical planning. It is recommended that biopsies be performed with awareness of the nail’s anatomic zones and with preoperative imaging if bone invasion is suspected [83].

### 7.3. Staging

While SCCNU is anatomically distinct from other cutaneous SCCs, it is currently staged using the UICC 9th edition (Union for International Cancer Control), the AJCC 8th edition (American Joint Committee on Cancer), the Brigham and Women’s Hospital (BWH) system, and the Breuninger/Tübingen staging system [84,85,86,87]. The T-stage definitions in the UICC and AJCC systems are primarily based on tumor size, with depth of invasion and PNI included as secondary risk factors [85]. The BWH staging system stratifies tumors based on the presence of high-risk features, which include: (1) tumor diameter ≥ 2 cm, (2) poorly differentiated histology, (3) PNI of nerves ≥ 0.1 mm, and (4) tumor invasion beyond subcutaneous fat. The T-stage in BWH increases with the number of these risk factors present. Unlike the UICC and AJCC systems, both the BWH and Breuninger systems are focused solely on local tumor characteristics and do not include N (nodal) or M (metastatic) staging components [84]. Those system provides a standardized framework for assessing tumor burden, local invasion, and metastatic spread, which are essential for prognostication, treatment planning, and research stratification [86]. Although the overall incidence of lymph node metastasis is relatively low—estimated at up to 5%—all patients should undergo thorough physical examination, with palpation of regional lymphatic basins as part of the initial assessment [72]. This clinical approach is generally adequate for patients with low-risk tumors. However, if regional lymphadenopathy is detected either clinically or via imaging, FNAC is recommended for diagnostic confirmation. For patients with primary SCCNU and no palpable nodes, routine imaging is not indicated unless the tumor exhibits high-risk features, as defined by the European Association of Dermato-Oncology (EADO) risk stratification criteria. In cases of advanced or high-risk SCCNU, comprehensive staging should be guided by a multidisciplinary tumor board, including input from a radiologist, to ensure appropriate selection and interpretation of imaging modalities [86].

## 8. Management and Prognosis

**Figure 3:**
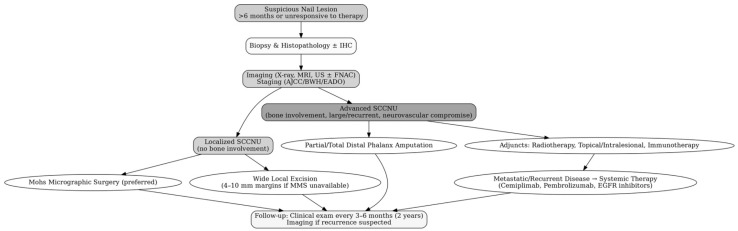
Treatment algorithm for squamous cell carcinoma of the nail unit (SCCNU). Stepwise clinical approach to SCCNU management. For localized disease without bone involvement, Mohs micrographic surgery is preferred; wide local excision is an alternative when MMS is unavailable. Advanced tumors with bone involvement or neurovascular compromise may require partial or total phalanx amputation. Adjuvant options include radiotherapy, topical or intralesional therapies, or immunotherapy in selected cases. Systemic therapy (e.g., PD-1 inhibitors and EGFR-targeted agents) is reserved for advanced or metastatic disease. All patients require structured follow-up with clinical exams every 3–6 months for two years, then annually.

The management of SCCNU is guided by several key factors: the tumor’s size, depth of invasion, histological grade, involvement of adjacent bone or soft tissue, HPV status, and patient-specific factors such as age, comorbidities, and functional expectations. The goals of treatment are complete oncologic clearance, the preservation of function and esthetics, especially of the digits, and the prevention of recurrence and metastasis. An individualized, multidisciplinary approach—often involving dermatologists, hand surgeons, pathologists, radiologists, and oncologists—is essential for optimal outcomes (Figure 3).

### 8.1. Surgical Management

Surgery remains the cornerstone of SCCNU treatment [2,88]. The choice of surgical modality depends on the extent of disease and available resources. Mohs micrographic surgery (MMS) is considered the gold standard for SCCNU when available, especially for tumors confined to the soft tissue without bone invasion [89]. MMS is a method of radical excision offering high cure rates due to the margin control and functional preservation. Studies have shown lower recurrence rates with MMS compared to wide excision or amputation, ranging from 78% to 96.5% [14,90,91]. Additionally, it allows precise excision in anatomically complex areas with maximization of tissue sparing while still achieving histological clearance, thereby decreasing patient morbidity and unnecessary amputations. When MMS is unavailable, wide local excision (WLE) with 4–10 mm margins is commonly employed. Delayed surgical complications, including epidermal inclusion cysts and nail spicule regrowth, occurred in 18% and 14% of patients [92]. Complete surgical excision of SCCNU with either WSE or MMS is associated with lower recurrence rates than limited excision and nonsurgical therapies, regardless of the degree of invasion [93]. Partial (functional) or total amputation of the distal phalanx is indicated in cases of bone involvement (confirmed by imaging or intraoperative findings), large recurrent tumors, failure of conservative surgery, or deep invasion with neurovascular compromise [94]. However, traditional amputations may result in functional problems, undesirable cosmetic results, and possibly psychological distress. This is particularly noticeable in the amputation of the thumb, which accounts for about 40% of the hand’s normal function. Further, the patient may experience loss of sensation at the amputated site, unusual pains, excessive sensitivity, and difficulties when gripping small items, which may interfere with daily tasks [95]. Regardless of the chosen surgical technique, the primary objective in the management of SCCNU should be the achievement of complete histological clearance, irrespective of whether bone involvement is present. Attaining this goal requires a comprehensive histopathological evaluation of all surgical margins.

### 8.2. Non-Surgical and Adjunctive Therapies

#### 8.2.1. Topical Therapies and Vaccination

While cryotherapy, topical agents, and photodynamic therapy (PDT) have been proposed as treatment options for SCCNU, their use remains limited due to several practical and clinical concerns [96]. These include the difficulty in delivering treatment effectively into the nail folds, the frequent requirement for nail plate avulsion in cases of subungual disease, and the lack of definitive histological confirmation of clearance [15]. Among topical agents, imiquimod has shown potential as an adjuvant therapy following surgical excision, although evidence remains limited [14]. More recently, therapeutic HPV vaccination has been explored as a novel treatment approach for HPV-associated cutaneous SCC. Specifically in the context of SCCNU, published case reports support the potential for HPV-directed immunotherapy as a noninvasive treatment strategy for HPV-related SCCNU [97]. However, the therapeutic role of HPV vaccination in SCC remains experimental. While evidence suggests that some elderly individuals can mount an effective immune response post-vaccination, further research is needed to determine whether higher or repeated dosing may enhance immunogenicity in older or immunocompromised populations. Ultimately, prospective, randomized clinical trials are necessary to establish the efficacy, optimal dosing, and HPV type-specific response to vaccination in SCCNU [3].

#### 8.2.2. Radiotherapy

Radiation therapy (RT) represents a viable treatment option for SCCNU in patients who are either medically ineligible for surgery or who decline surgical intervention. In one published case series involving 22 patients with nail unit SCC treated with RT, only one recurrence was reported. This recurrence occurred in an immunocompromised individual with polydactylous involvement and was associated with high-risk HPV infection [98]. It remains unclear whether the recurrence resulted from radio resistance induced by HPV or if it represented the emergence of new SCC foci. Interestingly, in contrast to this observation, HPV-associated oropharyngeal squamous cell carcinomas have demonstrated greater radiosensitivity than their HPV-negative counterparts. One proposed mechanism for this increased sensitivity involves the elevated expression of HPV viral transcripts, particularly E1–E4, which may enhance the tumor’s susceptibility to radiation-induced damage [99].

#### 8.2.3. Systemic Treatment

Although systemic therapies are not standard for localized SCCNU, they may be considered in cases of recurrent or metastatic disease according to published guidelines for invasive cutaneous SCC [100]. Specifically, Cemiplimab was approved for metastatic and locally advanced cutaneous SCC patients, who are not candidates for curative surgery or curative RT, whereas Pembrolizumab has not been approved by EMA in Europe, but it is FDA-approved since June 2020 [101]. Available targeted EGFR inhibitors (EGFRi) include antibody-based inhibitors of the extracellular domain of EGFR (cetuximab, panitumumab) and small molecule tyrosine kinase inhibitors (TKI), including erlotinib, gefitinib, and lapatinib [102]. Electrochemotherapy (ECT) has been used when all other treatment options, including surgery and RT, failed or were not feasible, if the patient refused any other treatments, and as palliative care to relieve symptoms, with limited evidence on the duration of local control and progression-free survival [103]. An emerging area of interest is the application of neoadjuvant immunotherapy [102,104,105,106,107].

This approach, already under investigation in high-risk cutaneous SCC and mucosal SCCs, involves administering immune checkpoint inhibitors (ICIs) such as anti-PD-1 antibodies (e.g., cemiplimab or nivolumab) prior to surgical resection [108]. The goal is to reduce tumor burden, potentially downstage the disease, and enhance surgical outcomes while preserving tissue and function [100,109,110]. In the context of SCCNU—where excision can compromise manual dexterity or require partial amputation—neoadjuvant therapy could play a transformative role by facilitating organ-sparing surgery. Although data specific to SCCNU are currently unavailable, early-phase clinical trials in cutaneous SCC have demonstrated high pathological response rates, even in immunocompetent patients [111].

### 8.3. Prognosis and Follow-Up

The prognosis for SCCNU depends on several variables:

- Tumor size and depth: larger and deeper tumors have a higher recurrence risk.
- Bone invasion is associated with poorer prognosis and the need for amputation.
- Histopathologic differentiation: poorly differentiated tumors as well as specific subtypes (e.g., desmoplastic, adenosquamous, and acantoholytic subtypes).
- PNI.
- HPV status.
- Surgical margin status.

Overall, SCCNU carries a good prognosis when diagnosed early. The cure rate exceeds 90–95% with appropriate surgical management, especially when performed via MMS. Prognosis worsens significantly with delayed diagnosis or inadequate excision. Recommended follow-up protocol includes clinical exams every 3–6 months for the first 2 years and imaging (ultrasound or MRI) if recurrence or bone involvement is suspected.

## 9. Future Directions

Early diagnosis remains the cornerstone of successful treatment, and emerging technologies such as videodermoscopy, reflectance confocal microscopy (RCM), and line-field confocal optical coherence tomography (LC-OCT) are showing promise in improving the detection of subclinical or in situ SCCNU [10,11,112,113]. These real-time, in vivo imaging modalities allow visualization of cellular and architectural abnormalities within the nail matrix and periungual tissues without the need for invasive biopsy. As their resolution and accessibility improve, they may facilitate earlier diagnosis, better margin delineation, and enhanced biopsy targeting, especially in lesions that are clinically ambiguous or anatomically challenging. Concurrently, advances in molecular characterization, particularly through next-generation sequencing (NGS), are providing new insights into the mutational landscape of SCCNU. While TP53 mutations are well-documented, broader panels are now uncovering rare but actionable alterations in genes such as EGFR, BRAF, HRAS, and DICER1, paving the way for targeted therapies [114]. In addition, the study of microRNAs (miRNAs)—small non-coding RNAs that regulate gene expression—is emerging as a promising avenue for both diagnostic biomarkers and therapeutic targets [115,116]. Specific miRNA signatures have been associated with squamous cell carcinogenesis, immune evasion, and response to therapy, and may soon be used to stratify patients by risk or predict treatment response in SCCNU [117,118]. Moreover, understanding the molecular differences between HPV-positive and HPV-negative tumors could guide the use of immunotherapies or virus-targeted strategies, including therapeutic vaccines. On the management front, the refinement of assisted surgical techniques such as robotic microsurgery, intraoperative margin mapping, and image-guided excision (e.g., RCM or LC-OCT–assisted) may further improve surgical precision and tissue conservation [80,119]. Innovations in intralesional therapy are being explored as minimally invasive options either as primary treatment in early disease or as adjuvant therapy to reduce recurrence. These approaches are particularly attractive in cases where surgery would result in functional or cosmetic compromise [120]. Ultimately, the convergence of early non-invasive diagnostics, molecular-guided therapy, and precision-based interventions holds great promise for transforming the clinical management of SCCNU—from a traditionally surgical paradigm to a multimodal, personalized treatment approach.

## 10. Conclusions

SCCNU remains a frequently misdiagnosed malignancy due to its subtle clinical presentation and overlap with benign nail disorders such as onychomycosis, paronychia, and warts. Its true burden is likely underestimated, compounded by diagnostic delays that may result in local tissue destruction, bone invasion, and in rare cases, metastasis. An accurate and early diagnosis hinges on a high index of suspicion, supported by clinical and dermoscopic examination, imaging evaluation, and histopathological assessment. Histopathology remains the gold standard, and its integration with immunohistochemistry and molecular tools can improve the comprehension of tumor behavior. Management of SCCNU should be individualized, with MMS being the preferred treatment in most localized cases due to its superior margin control and tissue preservation. In advanced disease, wide excision or amputation may be necessary. Adjunctive therapies, including topical agents, radiotherapy, and systemic treatments, are effective in some patients. Long-term follow-up is essential due to the risks of recurrence. Future research should aim to establish nail-unit-specific staging criteria, validate prognostic biomarkers, and explore targeted therapies informed by genomic profiling. Increased awareness and multidisciplinary collaboration will be key to reducing diagnostic delay and improving outcomes for this challenging yet treatable malignancy.

## Figures and Tables

**Table 1:** The following tablecompares SCCNU with other common nail conditions that are often mistaken for it, including onychomycosis, chronic paronychia, subungual wart, pyogenic granuloma, and amelanotic melanoma [22].

Condition	Common Age Group	Presentation Duration	Typical Symptoms	Diagnostic Clues
**SCC of the Nail Unit (SCCNU)**	50–70 years	Months to years	Pain, nail dystrophy, subungual mass, bleeding	Persistent lesion, irregular vascularity, poor response to therapy
**Onychomycosis**	All ages	Months to years	Discoloration, thickened nails, subungual debris	KOH positive, fungal culture, nail thickening
**Chronic Paronychia**	All ages (adults more common)	Weeks to months	Swelling, redness, tenderness around nail fold	History of wet work, inflammation without mass
**Subungual Wart**	Children and young adults	Months	Warty growth, rough surface under nail	Verrucous surface, HPV association
**Pyogenic Granuloma**	All ages	Acute (days to weeks)	Rapidly growing red nodule, bleeds easily	Bright red, lobular lesion, bleeds on contact
**Amelanotic Melanoma**	50–70 years	Months	Painless discoloration or subungual mass	Pigment network absent, vascular patterns
**Glomus Tumor**	20–40 years	Months	Severe localized pain, cold sensitivity	MRI: vascular nodule; intense pain on pressure

**Table 2:** Commonly observed dermoscopic characteristics of SCCNU, categorized by their underlying pathological basis.

Dermoscopic Feature	Description	Diagnostic Implication
Irregular vascular patterns	Dotted, glomerular, or serpentine vessels arranged chaotically	Suggestive of malignancy and neoangiogenesis
White/yellow hyperkeratotic masses	Compact keratin under the nail or surrounding nail fold	Correlates with tumor-induced keratinization
Onycholysis with jagged proximal edge	Separation of the nail plate with serrated inner margin	Common in both SCC and fungal infection
Hemorrhages	Red to black dots or streaks, often linear or globular	Indicates capillary rupture; not pathognomonic
Surface scaling	Roughened texture, often with debris accumulation	Frequently seen in verrucous SCC variants
Ulceration or crusting	Superficial erosion or scab formation over lesion	Indicates rapid growth or secondary trauma
Absence of pigment network	Especially important in amelanotic or pigmented lesions	Helps differentiate from subungual melanoma
Milky-red areas or polychromatic dots	Highly vascularized zones with mixed color features	Strongly suggests malignancy, especially in SCC

**Table 3:** Dermoscopic Differentiation from Common Mimickers.

Condition	Key Dermoscopic Features	Contrast with SCCNU
**Onychomycosis**	Linear white streaks, yellowish discoloration, uniform onycholysis	Lacks vascular irregularities or bleeding
**Wart (HPV-related)**	Papilliform surface with central black dots (thrombosed vessels), hyperkeratotic rim	More regular vascularity, less aggressive pattern
**Pyogenic Granuloma**	Homogeneous red area, white rail lines, collarette scale	Rapid onset and highly vascular but usually painful
**Melanoma**	Brown-black longitudinal streaks, Hutchinson’s sign, pigment network	SCCNU typically lacks consistent pigment features

## Data Availability

No new data were created or analyzed in this study. Data sharing is not applicable to this article.

## Abbreviations

The following abbreviations are used in this manuscript:

SCCNU	squamous cell carcinoma of the nail unit
HPV	human papillomavirus
RCM	reflectance confocal microscopy
LC-OCT	line-field confocal optical coherence tomography
IHC	immunohistochemistry
EMA	epithelial membrane antigen
NGS	next-generation sequencing
MRI	magnetic resonance imaging
FNAC	fine-needle aspiration cytology
PET-CT	positron emission tomography–computed tomography
AJCC	American Joint Committee on Cancer
BWH	Brigham and Women’s Hospital
PNI	perineural invasion
EADO	European Association of Dermato-Oncology
MMS	Mohs micrographic surgery
WLE	wide local excision
RT	radiation therapy
ICIs	immune checkpoint inhibitors
miRNAs	microRNAs
